# Performance characterization of a novel semi-active exoskeleton for overhead work

**DOI:** 10.1017/wtc.2025.10019

**Published:** 2025-07-30

**Authors:** Jonas Schiebl, Christophe Maufroy, Nils Ziegenspeck, Christof Giers, Bassam Elmakhzangy, Urs Schneider, Thomas Bauernhansl

**Affiliations:** 1 Fraunhofer Institute for Manufacturing Engineering and Automation IPA, Stuttgart, Germany; 2 Institute of Industrial Manufacturing and Management IFF, University of Stuttgart, Stuttgart, Germany

**Keywords:** performance characterization, mechatronics, industry, exoskeletons, design

## Abstract

Occupational shoulder exoskeletons can relieve workers during strenuous overhead work. Passive solutions are lightweight, robust, and cost-effective, but they can also restrict user movement, have limited support, and cannot dynamically adapt to different working conditions. Semi-active and active systems are still mostly the subject of research, and existing systems are heavy or have limited performance and support. Here, we present a lightweight semi-active exoskeleton for shoulder support that incorporates a novel motorized torque adjustment mechanism that varies the effective lever arm with which a spring applies force to the supporting joint. The mechanism is integrated into lateral structures and can be actuated via Bowden cables with motors located on the user’s back. The technical performance of the system was experimentally characterized in terms of its dynamic support torque profiles at six different support levels. Furthermore, adjustment times and energy consumption were investigated. The system showed plateau-like support torque profiles in the intended working range and could be adjusted from nearly 0 Nm up to 12 Nm of maximum support per arm. Adjustment times varied between 0.5 s for the adjustment of 20% of the total adjustment range and 1.0 s for a full activation/deactivation. Adjustments consumed between 0.1 As and 1.9 As of battery charge, allowing long operating times of up to one working day, using only a small 2 Ah battery. As a result, the exoskeleton provides high performance by combining comparatively high support, rapid motorized support adjustment, and low energy consumption in a lightweight design.

## 1. Introduction

Originally developed for military use, today’s exoskeletons are also used in medical or industrial applications. In medicine, exoskeletons help rehabilitate stroke patients or patients with neuromuscular impairments (Agrawal et al., 2017; Bogue, 2015; Gull et al., 2020). In industry, so-called occupational exoskeletons are introduced to relieve workers during heavy or repetitive work (Crea et al., 2021; Reyes et al., 2023). Their purpose is to prevent work-related musculoskeletal disorders (WRMDs) and the associated loss of productivity (Howard et al., 2020). WRMDs account for the majority of all self-reported work-related health problems in the EU (Kok et al., 2019) and are associated with high economic costs (Bevan, 2015; Kok et al., 2019). To date, occupational exoskeletons mainly support the upper extremities, particularly the shoulder during overhead work, or the back during bent-forward postures and lifting (Crea et al., 2021). In the following, we will discuss the challenges of occupational exoskeletons for shoulder support during lifting, carrying, and, in particular, overhead work.

Occupational exoskeletons can be further classified into passive, active, and semi-active exoskeletons (Reyes et al., 2023). Passive systems have a higher degree of maturity (Crea et al., 2021), and more passive than active commercial systems exist to date (see overviews in Crea et al., 2021; Gull et al., 2020; Reyes et al., 2023; Schiebl et al., 2022). Passive exoskeletons are more lightweight and cheaper than their active counterparts and are reliable and intuitive to use (Voilqué et al., 2019). Thus, passive systems are currently favoured in the occupational context (Voilqué et al., 2019). They contain passive energy storage devices (mainly springs) that are loaded or unloaded by the user‘s movement (Bär et al., 2021; Reyes et al., 2023). The resulting forces act on the human body so that the joints or muscles are relieved. A passive exoskeleton to relieve the shoulder during overhead work will, therefore, permanently push the user’s arm upwards and thus compensate for the weight of the arm and possibly the tool. The progression of the support torque depends on the flexion angle of the shoulder and is often designed in such a way that greater torques act at higher angles and decrease towards lower shoulder angles (Watterworth et al., 2023). Maximum support torques of commercial shoulder support exoskeletons vary between systems and exhibit values between 6 Nm and 13 Nm per shoulder (Musso et al., 2022; Pacifico et al., 2020; Watterworth et al., 2023). Investigations indicate that such devices reduce shoulder muscle activity in laboratory (Fritzsche et al., 2021) and field settings (Bock et al., 2020). To achieve greater biomechanical relief, higher support torques can be applied (Rafique et al., 2023), particularly when handling heavier loads, as demonstrated by simulations (Schiebl et al., 2022). However, the permanent upward support torque in passive systems requires the user to work against the system during downward movements. This can result in increased strain on the triceps, among other things (Van Engelhoven et al., 2018). This is acceptable with low support torques but will be increasingly exhausting with higher levels of support. To better adapt passive exoskeletons to different activities, some systems have manual adjustment options (Van Engelhoven et al., 2018; Watterworth et al., 2023). The fundamental advantage of such adjustment options was demonstrated in a study by Van Engelhoven et al. (2018). Users perceived different levels of support as ideal, depending on the activity, tool weight, or user anthropometry (Van Engelhoven et al., 2018). However, for this, one usually has to remove the system, which makes it difficult to adjust the support during work. Application-specific support seems important and modern workflows often involve dynamically changing tasks, so the adaptability of the exoskeleton to different working conditions is advisable (Crea et al., 2021). Hence, active exoskeletons could be a solution. They support users with active drives (mainly electric motors) whose support torques are continuously adjusted based on external information (e.g. sensor data) (Reyes et al., 2023). In contrast to passive systems, support torque profiles can be defined more flexibly (Voilqué et al., 2019) and adapted dynamically to different loads, theoretically even under highly dynamic working conditions (Crea et al., 2021). Active exoskeletons could potentially also provide stronger support (Grazi et al., 2020), as can be seen, for example, in the Stuttgart Exo-Jacket (up to 40 Nm per shoulder) (Ebrahimi, 2017). However, additional actuators and external energy sources and their control increase the complexity of the systems and make them heavier and more cost-intensive (Voilqué et al., 2019). The weight of the system is an important factor influencing wearing comfort and user acceptance (Elprama et al., 2022; Siedl and Mara, 2022), as users often report discomfort even with relatively lightweight passive solutions (~ 4 kg [Kim et al., 2022; Smets, 2019]). Frequently, the weight and torque-to-weight ratio remains unfavorable (e.g., Flexos: 5 kg, ~1 Nm/kg [Rinaldi et al., 2023]; Active Exo4Work: 10 kg, ~1 Nm/kg [Rossini et al., 2025]) when compared to passive exoskeletons (e.g., Paexo shoulder: 1.8 kg, ~7 Nm/kg [Maurice et al., 2020; Watterworth et al., 2023]; MATE: 3.5 kg, ~2 Nm/kg [Pacifico et al., 2020]).

Recently, so-called semi-active exoskeletons have been proposed as a technical compromise and potential next evolutionary step in exoskeleton development (Voilqué et al., 2019). Here, a fundamentally passive support mechanism is modulated by small drives to adjust the level of support (Grazi et al., 2020). This can result in systems that are more adaptable than passive solutions and at the same time compact and lightweight (Grazi et al., 2020). However, active and semi-active exoskeletons for the shoulder are still mainly subject of research and development efforts (overviews in Crea et al., 2021; Gull et al., 2020; Reyes et al., 2023) with only a few systems commercially available (e.g., AGADE, n.d.; ExoIQ GmbH, n.d.). An example of a semi-active (or “semi-passive”) exoskeleton for shoulder support is the H-PULSE, which can vary the preload of a torque-generating spring with a spindle motor (Grazi et al., 2020). In this way, support torques between 4.5 Nm and 6 Nm can be set in several stages (Grazi et al., 2020). The support level of the Lucy 2.0 pneumatic exoskeleton can also be adjusted up to a maximum of approximately 8 Nm (Sänger et al., 2022). Therefore, the maximum support torques of both systems have values comparable with low- to medium-strength passive shoulder exoskeletons (see Watterworth et al., 2023) but can additionally be adjusted during operation. Lucy 2.0 is characterized by the authors as an active system; however, it allows for various support levels, each with distinct angle-dependent support torque profiles generated by pneumatic cylinders (Sänger et al., 2022), which fundamentally function like springs. Consequently, we also categorize it as a semi-active system. The H-PULSE weighs 5 kg (Grazi et al., 2020), while Lucy 2.0 prototypes weigh approximately 5.5 kg (Otten, 2023), however, it remains unclear whether these weights include the battery. Nasr et al. also proposed a semi-active (or “active-passive”) exoskeleton based on the EVO exoskeleton (Nasr et al., 2023). This system can switch between three support levels, providing maximum torques of 5.6 Nm, 7.7 Nm, and 9.8 Nm. However, the working principle of the underlying mechanism was not described, and its weight remains unspecified (Nasr et al., 2023).

In conclusion, to address the disadvantages of passive exoskeletons – namely, their lack of adaptability to varying working conditions (Crea et al., 2021) – the next generation of exoskeletons could be active or semi-active. However, most fully active devices exhibit unfavorable torque-to-weight properties compared to passive systems and entail greater system complexity. To date, only a limited number of occupational semi-active shoulder support exoskeletons exist, with torque-to-weight ratios similar to active systems (~1 Nm/kg). These systems also demonstrate low maximum support torques (6–8 Nm [Grazi et al., 2020; Sänger et al., 2022]) compared to some passive solutions (up to 13 Nm [Watterworth et al., 2023]), even though increased support could be beneficial to mitigate biomechanical strain (Rafique et al., 2023), particularly as external loads become heavier (Schiebl et al., 2022). Systems need to be as lightweight as possible to allow for good user acceptance (Elprama et al., 2022), however, current solutions remain relatively heavy in relation to their functionality and maximum support. In (semi-)active systems, additional weight is required for the battery. Therefore, energy consumption directly influences the battery size and, consequently, the overall weight. Furthermore, one should be able to quickly adapt the exoskeleton to different working conditions during work, thus, adjustment speeds are an important performance metric as well. Yet, metrics for energy consumption and adjustment speeds have not been measured in the literature (Grazi et al., 2020; Sänger et al., 2022; Nasr et al., 2023; Otten, 2023) complicating direct performance comparisons.

In the present paper, we present a novel lightweight, semi-active exoskeleton that offers a high maximum support torque and can be rapidly adjusted to provide shoulder support for various tasks. Additionally, we propose methods to investigate its technical key performance metrics (support torques, adjustment times, and energy consumption) for future performance benchmarking. The exoskeleton’s basic mechanical design differs from the (semi-)active systems described above. With AGADE Exo, the Lucy 2.0 exoskeleton and the H-PULSE, two actuators (motors or pneumatic cylinders) are positioned next to the shoulder, generating a supporting torque to relieve the shoulder (AGADE, n.d.; Grazi et al., 2020; Sänger et al., 2022). A central back structure transfers the resulting forces and torques to a hip interface. Our approach, on the other hand, has no significant supporting central back structure. Two articulated, laterally positioned columns transmit forces and torques from the arm to the hip (see Section 2.2). This basic mechanical architecture is more similar to passive exoskeletons such as the Skelex 360 (Musso et al., 2022), the HAPO UP (ErgoSanté s.a.i., n.d.), or the Paexo Shoulder (Fritzsche et al., 2021), which positively influences freedom of movement (Maurice et al., 2020). Additionally, such designs have demonstrated significant biomechanical relief (Fritzsche et al., 2021; Maurice et al., 2020; Musso et al., 2022), even when working with heavy external loads (Schiebl et al., 2022).

Our approach integrates such architectures with a novel technology for motorized support level (SL) adjustment, enabling quick modulation of passive support torque between nearly zero and full support. This modulation is achieved by adjusting the distance of the supporting joint’s rotation axis from the vector of the acting spring force. In contrast, other semi-active approaches utilize the adaptation of spring pretensions (Grazi et al., 2020) or pneumatic pressure to modulate the acting force (Sänger et al., 2022). Our adjustment mechanism is located in the weight-bearing lateral columns connected to the hip attachment and is designed to be modulated by a force acting in only one direction (see Section 2.3). This allows spatially separated drives, located in a box at the back, to actuate the mechanism via Bowden tubes, which can only transfer significant forces in one direction (pull). Consequently, no drives or electronics are required in the lateral structures, resulting in lightweight and slim moving parts (see Section 2.1). Additionally, no electrical cable routing or housing is required, as all electronics are housed within the box at the back. The extra space in the back also facilitates the incorporation of further components in the drivetrain, such as reset springs and brakes (see Section 2.4). Reset springs assist in mediating the required maximum motor torques, allowing for the use of smaller and lighter drives. Permanent magnet brakes minimize overall energy consumption, as significant energy is only consumed during support level adjustments, enabling the use of small and lightweight batteries. All these measures contribute to a highly powerful system (with high support torques and rapid adjustment) relative to its overall weight.

Hereinafter, we describe the exoskeleton and its function in detail, before we examine key performance indicators for characterization in the second part. This includes an experimental investigation and analysis of the support torque profiles at different support levels (SLs), measurements of adjustment times, and energy consumption. Finally, we discuss experimental results and draw a conclusion on the performance of the exoskeleton.

## 2. System description

### 2.1. Overall design

**Figure 1.**
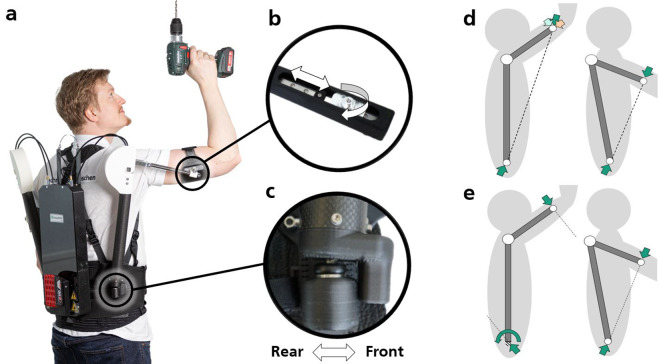
(a) User with semi-active exoskeleton. Two spring columns are laterally connected to a hip belt with a special joint that acts as a cardan or ball joint depending on the user’s shoulder flexion angle. Supporting forces are transferred to the arms via articulated arm bracings. A drive box containing the drive trains and all electronics is located at the user’s back. (b) Passive degrees of freedom (white arrows) in the connection to the arm interface, using a linear ball bearing with a carriage connected to the arm bracing via a revolute joint with plain bearings. (c) The special joint allows all rotational movements but blocks forward rotation above a certain flexion angle. (d) and (e) Schematic representation of the resulting forces and torques (green arrows) at the arm and hip interface of an exoskeleton at different shoulder flexion angles. (d) The ball joint at the hip interface, in combination with the revolute joint at the arm interface, leads to resulting force vectors, which, at large shoulder flexion angles, lead to high force components (orange arrow) parallel to the arm and thus to unpleasant shear forces (left figure). This effect is minimized at smaller shoulder flexion angles (right figure). The ball joint also allows the spring column to tilt backwards, allowing shoulder extension motions. e) If the special joint is used, it acts as a ball joint at low shoulder angles and allows a similar range of motion (right figure). However, at high shoulder angles (left figure), it locks and can also transmit torques, changing the direction of the reaction forces so that low shear force components occur in the arm interface.

The exoskeleton assists with lifting (from the waist up) and overhead work by providing perpendicular pressure to the upper arms from behind/below, to reduce glenohumeral torque and provide shoulder relief, similar to other systems with comparable architecture (Maurice et al., 2020; Fritzsche et al., 2021; Musso et al., 2022). External forces are transferred from padded upper arm bracings to the hip interface via arm levers connected to support structures, hereafter referred to as spring columns, which are laterally articulated to a standard hip belt (Figure 1). In addition to the hip interface, shoulder straps extend from the hip belt over the shoulders to stabilize the system on the body. Each lateral support structure contains a spring that applies a force to a lever mechanism, thereby generating a passive supporting force that pushes the arm upwards. An adjustment mechanism is also built into each spring column, which allows the support force to be modulated. A drive box on the user’s back transmits the required adjustment forces to the adjustment unit via Bowden cables. In addition to the drives, this box contains the control electronics and the power supply. The user adjusts the level of support using electronic rotary switches, which can be operated with two fingers. These switches can be affixed anywhere on the body using Velcro, such as on the shoulder straps, to ensure easy accessibility. The overall system weighs 5.4 kg without a battery and approximately 5.8 kg with a 2 Ah battery (GBA 18 V 2.0 Ah Professional, Robert Bosch Power Tools GmbH, Germany). The lateral structures (extending from the hip joint to the arm bracing) each weigh less than 1 kg and have a maximum width of less than 4.5 cm (at the thickest point, including the safety housing). The box has a thickness of 5.5 cm (excluding the battery). The following subsections describe the implementation of the mechanical structure, including its actuators, drives, and electronics.

### 2.2. Kinematic structure

**Figure 2.**
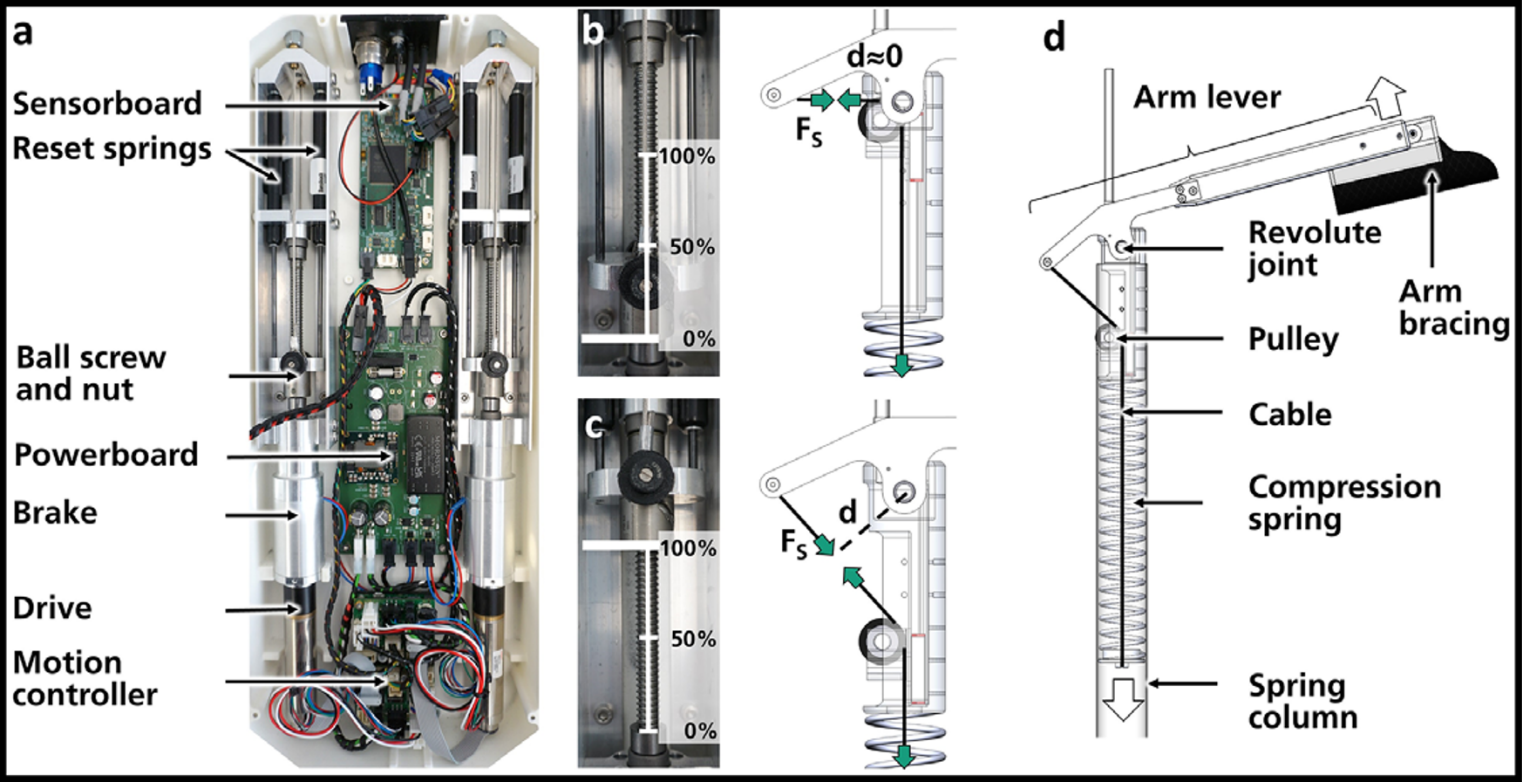
(a) Drive box with two drive trains (drives, brakes, ballscrews and nuts, reset springs) controlled by motion controllers and a sensor board powered by a power board connected to a battery (not shown). (b) and (c) ballscrew nut positions (left) and corresponding positions of the pulley in the adjustment unit (right). The examples show complete deactivation (b), when the lever arm d is approximately zero, and full activation (c), where the lever arm d is significantly larger, resulting in the highest supporting torque. Cable/Spring forces *F*_s_ (cut free) are shown as green arrows. (d) Working principle of the torque generation. A compression spring in the spring column pulls on a cable downwards (white arrow), which is connected to the rear end of an arm lever via a pulley, causing the arm lever to rotate around the revolute joint and the arm bracing to be lifted (white arrow) or support forces to be applied to the arm.

At shoulder level, each spring column has a spring-actuated revolute joint (plain bearing) supporting the shoulder flexion/abduction movement (hereinafter referred to as flexion for simplification). The resulting force acting on the upper arm is generated by a lever mechanism, whereby a spring is used to pull the arm lever down on the arm’s opposite side to the revolute joint with a cable (Figure 1[a] and Figure 2[b]). The arm bracing is connected to the arm lever via another revolute joint to minimize torques in the arm interface (Figure 1[b]). These torques are difficult to avoid, as the dynamic movement of the shoulder (elevation, etc.) during work always results in a certain misalignment between the rotational axes of the shoulder and the exoskeleton. The torques manifest in the form of concentrated compressive forces on the skin in the area of the edges of the arm bracing, which can become uncomfortable at higher support torques (Jarrasse and Morel, 2012). To avoid shear forces in the arm interface, a linear joint providing a translational degree of freedom along the arm lever is also implemented using a linear ball bearing (Figure 1[b]).

A special joint is installed in the transition to the hip belt (Figure 1[c]), which, depending on the shoulder flexion angle, has two or three rotational degrees of freedom (DOF), that is, it acts either like a cardan or a ball joint. This design aims to facilitate a high range of motion (ROM) while simultaneously minimizing shear forces at the arm interface. At high shoulder flexion angles, the combination of the ball joint in the hip and the revolute joint in the arm interface often leads to uncomfortable shear forces in the arm and, consequently, to slipping (Figure 1[d]). The special joint offers a rotational degree of freedom to the rear, side, and the spring columns’ longitudinal axis, allowing the lateral spring columns to move backwards at low shoulder flexion angles, for example, when the user places his or her arms next to the body (Figure 1[e]). However, the special joint locks at high shoulder flexion angles, allowing it to transfer not only forces but also torques (Figure 1[e]). As a result, the kinetics of the system change, and shear forces in the arm interface are minimized. Since the hip belt alone cannot comfortably absorb all of the resulting torque, part of the torque is transferred to the upper back in the form of perpendicular forces via a linkage. The drive box, which contains all the drive and control components, is also attached to this linkage (Figure 1[a]).

### 2.3. Adjustment unit

Inside the spring columns are pre-tensioned compression coil springs (custom-made: dxDMxL0xnxWNR = DF3x33x570x36xSH, Gutekunst + Co.KG, Germany) that pull on a cable, which is guided over a pulley (plain bearing) and then engages at the rear end of the arm lever (Figure 2[d]). The normal distance between the revolute joint and the cable (imaginary lever arm), in combination with the acting cable pull force, generates torque around the revolute joint. A corresponding counteracting support force in the arm interface achieves the torque balance in the revolute joint. In the following, we refer to the torque as a reference value for the support. This reference is independent of the variable distance between the revolute joint and the arm connection. When the arm moves downwards, the spring is tensioned, which increases the cable force. At the same time, the imaginary lever arm changes over the shoulder flexion angle, resulting in a characteristic angle-dependent support torque curve. This profile can be changed by additionally limiting the maximum distance between the revolute joint and the cable using the adjustment mechanism. For this purpose, the pulley is located on a ball-bearing carriage, which can be positioned along a rail in the spring column. Parts of the cable forces on the carriage are directed in parallel to the rail. A suitable geometrical design of the mechanism and its location in the spring columns (instead of the arm levers, like in similar passive system architectures, see [Maurice et al., 2020; Musso et al., 2022]) ensures that the resulting force vector acting on the carriage points away from the revolute joint at any flexion angle and level of support. This presents the advantage that a unilateral force transmission element, such as another cable, can be used to position the carriage, acting against the resulting force pushing the carriage away from the revolute joint. In the present implementation, we use Bowden tubes and cables for that purpose (spiral sheath SP000002 with wire LI000046, Carl Stahl Technocables GmbH, Germany), which are actuated via remote drive units located in the drive box at the back (Figure 2[a]). In this way, the supporting torque increases as the carriage moves away from the revolute joint (Figure 2[c]) and decreases (to virtually zero) as the motor pulls it towards the joint (Figure 2[b]).

### 2.4. Drives and electronics

The drive unit consists of an electric motor (EC-4pole 22, brushless, 120 W, 24 V and planet gearbox GP 22, transmission: 4.4:1 and Encoder 16 EASY, 1024 counts per turn, maxon motor GmbH, Germany) with a holding brake (permanent magnet brake 86 61103H00-0005, Kendrion (Villingen) GmbH, Germany) that drives a ball screw (Carry KGT 8x2 FGR RH, diameter: 8 mm, pitch: 2 mm, Eichenberger Gewinde AG, Switzerland) and thus translates the drive torque into a force on a ballscrew nut (Figure 2[a]). This pulls on the Bowden cables used in the adjustment mechanism via an adapter, thus defining the acting force’s distance to the revolute joint (Figure 2[b] and [c]). Two reset springs (gas pressure springs, Q0Q0-0P-070-166/110 N, Bansbach easylift GmbH, Germany) press on the same adapter. Without these springs, the drive would only have to apply force in one direction of movement, namely to pull the carriage in the adjustment unit closer to the revolute joint. In the other direction, the movement occurs passively (see Section 2.3). The reset springs ensure that the drive has to apply higher forces when moving the pulley away from the revolute joint and lower forces when moving towards the joint. The springs are dimensioned so that the maximum forces required in both directions are theoretically similar and significantly lower overall than the maximum force required without springs. This means that less powerful, smaller, and lighter motors can be used. The holding brake is a permanent magnet brake only opened during the adjustment process. After the adjustment, it closes and prevents the SL from being changed even under maximum external load. Hence, the motor requires no holding current, which minimizes power consumption and allows the use of smaller batteries. The control and supply electronics are located in the drive box together with the drive trains. A power board is powered by a power tool battery (Figure 2[a]). This, in turn, powers the motion controllers (EPOS4 Compact 50/5 CAN, maxon motor GmbH, Germany) of the motors, the holding brakes, and a sensor board (Figure 2[a]). The latter receives external sensor signals and uses them to control the electromechanical components. In this case, the system is equipped with a pushbutton for activating and deactivating the assistance and with a rotary switch on which the degree of assistance can be varied in six stages (0%, 20%, 40%, 60%, 80%, 100%). It should be noted that these levels have been chosen at random for the tests described below. Theoretically, quasi-continuously adjustment is also possible. In the following chapters, we present the methods and results of characterization experiments for our latest prototype, which has been optimized for overhead work.

## 3. Characterization methods

### 3.1. Experimental setup

**Figure 3.**
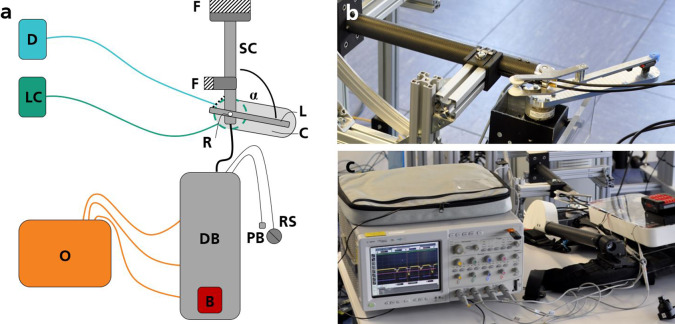
(a) Drawing of experimental setup with one exoskeleton spring column (SC) fixed to a frame (F). The arm lever (L) is connected to a cantilever (C) while the axis of the revolute joint (R) is aligned concentrically to the axes of the load cell (green, LC) and external drive (blue, D), each connected to the corresponding control infrastructure. The drive box (DB) uses energy from a battery (red, B), and selected electronics components are connected to an oscilloscope (orange, O). The SL can be varied using a pushbutton (PB) or rotary switch (RS). The angle α is varied during experiments. (b) and (c) Photos of said components of the experimental setup. (b) Fixed exoskeleton arm with cantilever, load cell, and external motor. (c) Oscilloscope connected to the drive box.

**Figure 4.**
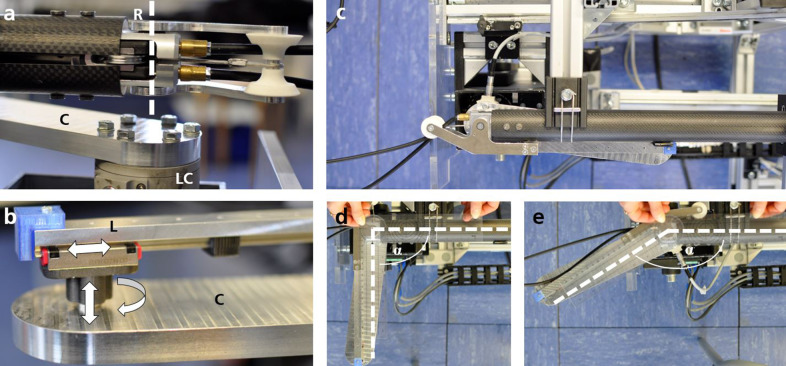
(a) Exoskeleton revolute joint (R) arranged concentrically (white dashed axis for visualization) to load cell (LC) connected to cantilever (C) and external drive. b) End of the arm lever (L) connected to the cantilever (C) via revolute joint and linear ball bearing. White arrows indicate DOFs in the connection. (c), (d), and (e) Experimental setup from above with the arm lever in parallel to the spring column (c) and with exemplary angles of *α* = 90° (e) and *α* = 150° (e), visualized with dashed white lines and a goniometer.

An experimental setup was created to determine the exact support torque profiles, adjustment times, and power consumption of the system (Figure 3). For this purpose, the lower end of the right spring column of the exoskeleton was attached to a rigid frame using an adapter. The spring column and arm lever were aligned in parallel to the floor (Figure 3[b]). To prevent the spring column from shifting during the tests, a rigid frame also supported the top of the column (Figure 3). Visual inspection and a spirit level (Laserliner, UMAREX GmbH & Co. KG, Germany) ensured the correct positioning and orientation of the components before testing and regularly during the tests. A powerful electric motor with gearbox (EC 45 brushless, 250 W, 48 V and planet gearbox GP52C, transmission: 43:1 and Resolver Res 26, maxon motor gmbh, Germany) was arranged under the spring column and rigidly connected with a load cell (6-axis force-torque sensor K27 x 53, SENSIX S.A.S., France) concentrically to the drive axis (Figure 4[a]). A cantilever was rigidly connected to the other end of the load cell and led to the end of the arm lever (Figure 4[a] and [b]). There, it was connected via a revolute joint to a linear ball bearing (MINIRAIL 1-MNNL-7-G3 Schneeberger GmbH, Germany) on the arm lever (Figures 1[b] and 4[b]). The connection ensures torque-free transmission of forces. In addition, small relative displacements in the vertical direction and the longitudinal direction of the arm lever are possible without constraining forces occurring. The revolute joint axis of the supporting exoskeleton joint was aligned concentrically to the axis of the external drive and the load cell. All axes were, therefore, vertical to the ground, and the plane of movement of the arm lever was parallel to the ground, which meant that gravitation could not influence the measured support torque profiles. We checked the concentricity of the axes using a ruler to measure the maximum relative displacement between the cantilever and the arm lever during a full movement of the arm lever from the lowest to the highest flexion angle. This was never more than 2 mm in the vertical direction or along the linear ball bearing. In addition, preliminary tests were carried out to ensure that no relevant constraining forces occur in the load cell due to relative displacement. Load cell signals were recorded with Qualisys Track Manager (QTM) 2023.1 (Qualisys AB, Sweden). The external drive was controlled using the Software Elmo Application Studio (EAS) II (Version Number 2.8.0.22, Elmo Motion Control Ltd., Israel) via a motion controller (Platinum Twitter Solo, Elmo Motion Control Ltd., Israel). An oscilloscope (Infiniium 8000 Model MSO 8104A, Agilent Technologies, Inc., CA, USA) was used to measure the adjustment times and voltages (Figure 3) at various electronic components of the exoskeleton, from which the power consumption was calculated.

### 3.2. Support torque profiles

At the start of the measurements, the external drive was rotated until the arm lever was almost parallel to the spring column and this alignment was defined as the starting point (Figure 4[c]). The angle between the spring column and the arm lever at the starting point was determined with a goniometer (Luckaide, China) at approximately 1°, and all angles were corrected by this amount later. Then, each measurement was carried out as follows: the arm lever and cantilever were separated and the load cell was reset. As a result, the weight forces of the cantilever and the resulting moments in the load cell are not included in the evaluation. One of six different SLs (0%, 20%, 40%, 60%, 80% and 100%) was set on the exoskeleton. The arm lever and cantilever were then reconnected (Figure 4[b]), and the arm lever was moved to the start position (Figure 4[c]). The arm lever was then rotated from the start position to an end position (at approx. 155°) at a speed of 10°/s using the external drive. This movement is also referred to below as flexion (corresponding to shoulder flexion motions). Then, the arm lever was rotated back to the start position at the same speed, referred to below as extension. This cycle was repeated five times. The entire procedure was repeated for all six SLs and again at a rotation speed of 50°/s to determine whether speed-dependent behaviour occurred. Signals from the load cell were recorded and stored using QTM. In addition to load cell measurements, the motor position of the external drive, its speed, acceleration and current were recorded and saved for each measurement using EAS II.

### 3.3. Adjustment times and energy consumption

In addition to the support torque, the time required to adjust the system is an important performance parameter. To measure this, the same mechanical test setup was used as described above. In order to emulate the influence of different external arm postures and loads during adjustment, different flexion angles (0°, 30°, 60°, 90°, 120°, 150°, see examples in Figure 4[c]–[e]) were set one after the other with the aid of a goniometer and motion was confined with the activated external drive. Electrical voltages were recorded in a time-synchronized manner between the battery and the power board, at the push button, the rotary switch, and the brake. For this purpose, the oscilloscope was used with a sampling rate of 1000 Hz. The total battery current was calculated from the voltage measured with the oscilloscope across a 0.1 ohm resistor. During all measurements, the unused drive and brake (left side) were disconnected from the motion controller/power board. The voltages and adjustment times were recorded as follows: An angle was set, and the exoskeleton was activated. For each measurement, a start and stop SL was defined from one of 15 possible combinations (0%–20%, 0%–40%, 0%–60%, 0%–80%, 0%–100%, 20%–40%, 20%–60%, 20%–80%, 20%–100%, 40%–60%, 40%–80%, 40%–100%, 60%–80%, 60%–100%, 80%–100%). The measurement was started on the oscilloscope, and the SL was adjusted five times from the start SL to the stop SL and then back again by quickly turning the rotary switch manually to the corresponding position and then turning it back to the start. During adjustment, the control algorithm automatically opens the brake first, then the drive makes the adjustment motion, and the algorithm closes the brake again. The measurement was saved and repeated for the next SL combination. The procedure was carried out for all six angles so that 90 adjustment measurements were taken, each with five repetitions. After each set, a goniometer was used to check whether the set initial angle was still correct. In addition, a measurement was carried out in which the pushbutton or switch voltage and the brake voltages were explicitly recorded to measure the time between pushbutton/switch and brake activation. Furthermore, a measurement was carried out in which all motors and brakes were disconnected from the power supply to record the electronics’ basic power consumption (motion controller, sensor board, power board).

### 3.4. Data processing and analysis

Data processing and evaluations were carried out using MATLAB R2023b (The MathWorks, Inc., MA, USA). For support torque profile investigations, the signals of the load cell (see Section 3.2) were translated into forces and torques using an associated calibration matrix. The torque around the axis concentric to the revolute joint’s axis was extracted for further evaluation, and signal jitter was reduced using a Butterworth low-pass filter (order: 2, cut-off frequency: 5 Hz) without visibly changing the signal shape. Finally, the torque signals were time-aligned with the drive data (current signal of the motor) so that angle-dependent torque profiles could be derived from the angle data of the drive and the torque data of the load cell and resulting profiles were averaged across the five repetitions.

For the analysis of adjustment times (see Section 3.3), unprocessed voltage signals were analyzed to achieve the most accurate results possible. The time difference between the onset of the switch or pushbutton signal and the brake signal was calculated and averaged over five repetitions. Furthermore, the time between activation of the brake at the beginning and deactivation of the brake at the end of the adjustment process was calculated for the first four movements from a lower to a higher SL (hereinafter called activation) and for the first four movements from a higher to a lower SL (hereinafter called deactivation). Times for activation and deactivation were averaged separately over the four repetitions and determined for all 75 adjustment measurements. The signal noise of the calculated battery currents was minimized using a Butterworth filter (low-pass filter, order: 2, cut-off frequency: 10 Hz). For energy consumption analysis (see Section 3.3), the consumption of electrical charge per adjustment was determined by integrating the current over time (MATLAB function: *trapz()*), with the activation (permanent magnet brake is loose) of the brake marking the beginning and the deactivation of the brake marking the end of the adjustment process. Before that, the electronics base current was subtracted from the current determined for each individual adjustment measurement so that a distinction could be made between the consumption of the actuator unit and the consumption of the electronics. Charge quantities were averaged over four repetitions per parameter combination, whereby a distinction was made between activation and deactivation movements.

In addition to graphical analyses, regression models were generated in the statistical software Minitab (Minitab GmbH, Germany) to identify relations between start and stop SL, flexion angle (independent variables), and resulting adjustment times or charge quantities consumed (dependent variables). Linear regression models were used for both target variables (adjustment time and charge consumption). Linear models were insufficiently accurate for charge consumption determination, so additional regression equations were formed in which terms and interactions up to the third order were permitted. Non-significant terms (*p* > 0.05) of the regression equations were successively removed, starting with higher-order terms, to minimize the complexity of the equations. Finally, also significant terms (*p* < 0.05) with the highest *p*-values were removed as long as the resulting *R* ^2^ of the regression equation was greater than 99%. Finally, the regression models were validated using the fifth repetition of each measurement, which had not been used previously. For this purpose, the percentage deviation between the model and the measured values was calculated for all parameter combinations.

The higher-order regression equations were used to estimate the operating time of a battery with 2 Ah electrical charge for three fictitious cases. For each case, the number of times the system is adjusted per minute was defined, together with the start and stop SL. The working angle at which the adjustments take place was set at 90° for all cases to simulate generic overhead work. To simplify matters, we assumed that both sides of the exoskeleton always undergo the same adjustment. The regression equations were used to calculate the amount of charge consumed by the drive and battery for activation (*Q*_A_) and deactivation (*Q*_D_), doubled (two exoskeleton arms) and multiplied by the number of adjustments per minute (*N*_60_) (see equation 1). The charge consumed by the base current of the electronics (*Q*_EL_) was added to determine the total charge consumed per minute. Then, an operating time (*t*) was calculated using the battery charge (*Q*_Bat_).
(1)

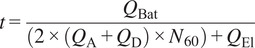



The three fictitious cases were:

- **Case 1 – Construction site:** infrequent (every 5 min) but complete activation and deactivation (0% - 100%), for example, to fetch material, pick up tools or take a short break.
- **Case 2 – Assembly line:** closely synchronized work steps with tools of different weights. The system switches from 40% to 80% support 12 times per minute.
- **Case 3 – Quasi active:** The system is adjusted by one SL every second (here 80%–90% was specified) to always provide the exact level of support required in an automated support control approach.

## 4. Results

### 4.1. Support torque profiles

**Figure 5.**
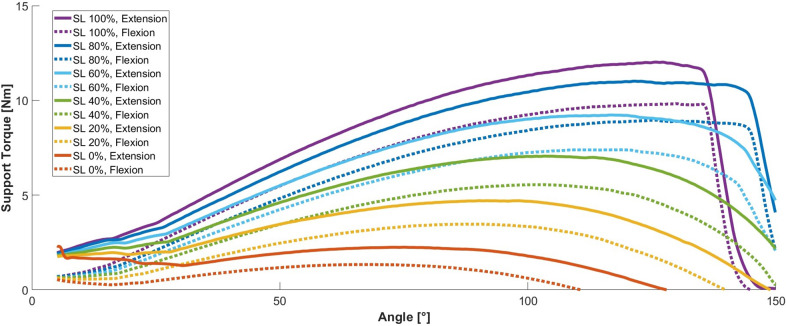
Mean support torque profiles (averaged over five repetitions) over flexion angle for different SLs (0%–100%) and for flexion (dotted line) and extension (continuous line) movements. Values rise to a maximum (between 68° and 130°) and then fall back to zero afterwards. Note that the purple line (100% SL) falls back at a smaller angle. Values of torque maxima and corresponding angles are presented in Table 1.

*Figure 5* shows the mean support torque profiles as a function of the different SLs and directions of movement (velocity: 10°/s). The curves start at 5°, as interactions with a small safety cushion at the spring column distort the results unrealistically near the zero point. The exoskeleton supports each shoulder with a mean maximum torque of up to 12 Nm. Depending on the angle, the torque curves rise gently at higher flexion angles until they reach a maximum, from which they fall again. The profiles result from the acting spring force and the distance between the revolute joint and the pulley position (can be adjusted, as shown in Figure 2), which varies with the flexion angle. At high flexion angles in combination with the highest SLs, the torque decreases abruptly due to mechanical end stops (Figure 5). Below 30°, the profiles deviate from their monotonic progression (as indicated by small inflections in the profiles, see Figure 5), which is caused by unintended mechanical interference of the arm lever with the Bowden tubes (see Figure 4[c]). Around the maximum, the support is almost constant in large angular ranges to provide the most intuitive support feeling possible. The plateau-like areas include a range of at least 31° in which the support torques deviate by less than 10% from the respective maximum (Table 1). At higher degrees of support, the ranges tend to become wider (up to 55°).

**Table 1.** Maxima of mean support torque profiles for six different SLs for flexion and extension movement directions. The angles at which the maxima occur and the angular range in which the support torques deviate by less than 10% from the respective maximum are shown in the second and third columns for flexion and extension.

	Extension			Flexion		
Support level	Max. torque [Nm]	Angle [°]	Range [°]	Max. torque [Nm]	Angle [°]	Range [°]
0%	2.2	75	37	1.3	68	31
20%	4.7	90	45	3.5	88	39
40%	7.1	103	49	5.6	103	45
60%	9.2	117	54	7.4	117	50
80%	11.0	121	55	9.0	126	53
100%	12.0	126	45	9.8	130	45

Also, the SL influences the torque profiles. An SL reduction shows significant reductions in the support torque. Their maxima decrease almost directly in proportion to the SL. At an SL of 0%, however, maximum torques of 1.3 Nm (flexion) and 2.2 Nm (extension) still apply (Table 1). Furthermore, a reduction in the SL not only reduces the amount of angle-dependent torque but also shifts the angle of the maximum to lower values, from approx. 126° at 100% (extension) or 130° at 100% (flexion) down to 75° at 0% (extension) or 68° at 0% (flexion). At higher SLs, the support decreases more and more steeply from a certain angle. At the highest SL, the support torque falls towards zero at a slightly lower angle. Another relevant effect is that the support torques reach zero earlier at low SLs (e.g., 0%: ~110°). The direction of movement influences the torque profiles, too. The system supports movements in the flexion direction with up to 9.8 Nm, movements in the opposite direction with up to 12.0 Nm per arm (Table 1). The torque differences get higher as the SL increases. The absolute difference between the flexion and extension movements rises from 0.9 Nm to 2.2 Nm, while the relative torque reduction falls from 41% to 18%.

Furthermore, we investigated the influence of different velocities on the support torque profile. Standard deviations between the averaged torque profiles from 10°/s and 50°/s were determined with otherwise identical parameter combinations (direction of movement, SL). The motion velocity had very little influence on the torque, with a standard deviation averaged over the torque profile and all parameter combinations of 0.06 Nm. As expected, the support torque was slightly reduced for flexion movements at higher speeds but increased for extension movements. The repeatability of the measurement method was quantified as well: Differences in the torque curve of the five measurement repetitions (with the same parameter combinations) were small across all six SLs, both directions of movement and both velocities. The standard deviation over the five repetitions of a parameter combination was determined for each point of the torque curve. The standard deviations averaged over the torque curve and the 24 parameter combinations (six SL, two directions, two velocities) amounted to 0.01 Nm.

### 4.2. Adjustment times and energy consumption

**Figure 6.**
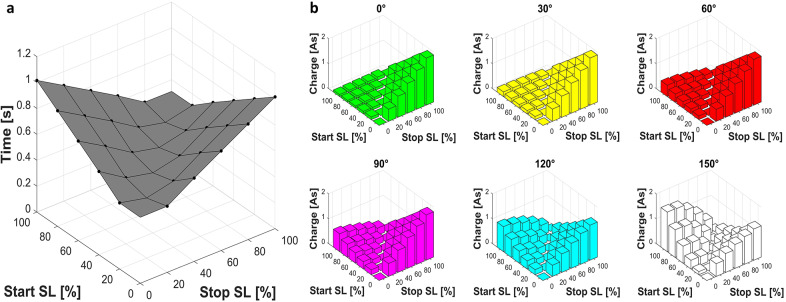
(a) Adjustment times when moving from start to stop SL, averaged over four repetitions for each parameter combination. (b) Each bar plot shows the mean charge consumed by the brake and the motor as a function of start and stop SL. Different diagrams show the results for different flexion angles α. The left part of all diagrams (a and b) shows deactivation movements (reduction of the SL), and the right side shows an increase in the SL.

The times between the pushbutton or potentiometer signal and the brake signal were on average 7.5 ± 0.7 ms and 5 ± 0.8 ms, respectively. The adjustment time, that is, the time between opening the brake before and closing it after the movement, varied depending on the adjustment distance. Figure 6(a) shows the adjustment times during the transition from start to stop SL, averaged over four repetitions for each parameter combination (standard deviations, averaged over all measurements: 0.5 ms). It can be observed that the activation and deactivation movements take approximately the same time and follow a linear trend. Therefore, it makes no difference whether the SL is increased for example, from 0% to 60% (0.77 s) or reduced from 60% to 0% (0.77 s). Higher distances between start and stop SL require longer adjustment times of up to 1.02 s for adjustments between 0% and 100%. Further statistical analyses underline these results. Regression analyses with Minitab showed that the flexion angle of the exoskeleton had no relevant influence (*p* > 0.05). Regression analyses with only the predictors start and stop SL resulted in equations that could predict the adjustment times with high accuracy (coefficient of determination *R* ^2^ > 99.9%). The coefficients in the regression equations for activation and deactivation were equal (Table 2). The minimum duration of 0.386 seconds can be explained by motor acceleration and deceleration times. The deviation of the validation measurement from the calculated model values was 0.4% on average (maximum 0.8%) for both activation and deactivation.

**Table 2.** The table shows regression equations to estimate adjustment times and consumed charges for activation (Act.) and deactivation (Deact.) movements, depending on the start and stop SL (SL_Start_ SL_Stop_) and the flexion angle (*A*_f_). For the adjustment time, a linear regression model is given, for the charge calculation, a linear (simple), and a model of higher polynomial order (precise) is shown. On the right, coefficients of determination (*R* ^2^) for the equations can be found as well as average deviations of the calculated model values from an independent measurement.

Parameter	Process	Equation	*R* ^2^ [%]	*D* [%]
Time	Act.	0.386 + 0.635(SL_Stop_ – SL_Start_)	> 99.9	0.4
	Deact.	0.386 + 0.635(SL_Start_ – SL_Stop_)	> 99.9	0.4
Charge (simple)	Act.	–0.175A_f_ − 1.492SL_Start_ + 1.850SL_Stop_	98.7	13.0
	Deact.	0.439A_f_ – 0.686SL_Start_ + 0.320SL_Stop_	88.4	59.3
Charge (precise)	Act.	−1.8227 SL_Start_ + 1.9305 SL_Stop_ + 0.4230 SL_Start_ A_f_^2^–0.4364 SL_Stop_ A_f_^2^ + 0.1709 SL_Stop_^2^ A_f_	99.8	6.0
	Deact.	0.1164S L_Stop_ + 0.6884 SL_Stop_ A_f_ + 0.0603 A_f_^3^ + 0.4120 SL_Start_^2^ A_f_ – 1.0599 SL_Start_ A_f_^2^–0.6420 SL_Stop_^2^ A_f_ + 0.5389 SL_Stop_ A_f_^2^	99.3	16.8

The charge consumed by the motor and the brake was calculated for each adjustment and averaged over the four repetitions (standard deviations averaged over all measurements: < 1 mAs). Figure 6(b) shows the charge depending on flexion angle, start SL, and stop SL. At an angle of 0°, little charge is required for deactivation movements, but more and more charge is required for activation with increasing adjustment distance. As the flexion angle increases, the required activation charge tends to decrease slightly, but the charge required for deactivation increases. The adjustment distance also has an increasing effect on the deactivation charges as the angle increases. This results in total charges of 0.1 As to 1.6 As for the deactivation and 0.3 As to 1.9 As for the activation movements. The diagrams also show that the charge required for activation increases almost linearly as a function of the displacement, while higher-order polynomials must be used to describe deactivation. Regression analyses were performed in Minitab to better understand the relationship between angle, start/stop SL (independent predictors), and activation/deactivation charge better. The regression models can be found in Table 2. The linear regression models could already explain the consumption of the activation charge well (*R* ^2^ = 98.7%), but the deactivation functions were not very accurate (*R* ^2^ = 88.4%). Therefore, higher-order regression equations were set up, showing higher coefficients of determination (*R* ^2^ > 99%). The average deviation of the model values from the fifth repetition measurement was 6% for activation and 17% for deactivation. Based on the higher-order regression equations and the base current of the electronics (0.28 A), the theoretical operating time of a 2 Ah battery was calculated for three predefined fictitious cases (see Section 3.4). This was 6.8 h for case 1 (construction site), 4.1 h for case 2 (assembly line), and 2.2 h for case 3 (quasi-active).

## 5. Discussion

### 5.1. Support torque profiles

The experiments demonstrate that the exoskeleton can generate varying support torques as desired, achieving a maximum support torque of 12 Nm. The support torque profiles align with our expectations based on idealized mechanical models. However, unintended effects (e.g., friction and mechanical interferences) have slightly influenced the magnitude and course of the curves, which will be discussed in further detail later. The change in support torque around the maxima is relatively small over wide angular ranges. We assume that the plateau-like support in the working range feels intuitive and makes working comfortable. However, this behaviour is not new per se; other systems show similarly small changes in the support torque around the maximum (Watterworth et al., 2023). What is new, however, is the mechanism for motor-driven SL adjustment. The mechanism scales the support torque almost directly proportional to the SL, which can facilitate control. It is also possible to reduce the support torque to very low values (maximum torque at SL 0% is smaller than 20% of the maximum torque at SL 100%), which minimizes the system’s motion resistance when deactivated. In contrast to other passive systems (Watterworth et al., 2023), in our mechanism, an adjustment of the SL is not only accompanied by a change in the torque but also by a shift in the working range. From our point of view, this can be advantageous since the user can select lower SLs when carrying out activities that require a lower shoulder flexion angle and higher support for overhead work with high shoulder flexion angles, which are associated with shoulder complaints, especially in combination with external loads (Bernard and Putz-Anderson, 1997). In addition to these general findings, there are some effects in the results that require further explanation which are discussed below.

There are clear differences in the support torques of flexion and extension movements. These are probably caused by friction in the system, particularly in the pulley and the revolute joint, and in the contact between the spring and the spring column. During flexion, the movement relaxes the spring, which pulls the cable inwards over the pulley. Friction in the pulley (plain bearing) reduces the cable force that generates the support torque. The reverse is true in the opposite direction. Tensioning the spring causes frictional forces to act in the same direction as the spring force. The friction in the pulley additionally increases the force that must be applied to the cable from the outside. The efficiency of our pulley was mathematically estimated using geometric data and approximate friction coefficients. Potential friction losses were found to be up to 8% for a friction coefficient of 0.1. The resulting potential difference between the flexion and the extension movement of 16% can already explain a major part of the relative deviations we found in the experiments (18–41%). In addition, there is friction in the revolute joint, which, however, depends only on the resulting forces of the arm lever and is not influenced by the support torque-generating lever arm length. Therefore, it is more independent of the SL, which explains the absolute torque difference of 0.9 Nm at SL 0% and the associated higher percentage deviations at small SLs. Friction losses due to the contact between the spring and the spring column are estimated to be low (given the size of the other sources of friction) and expected to be lower than with gas springs (which already have high internal friction). Therefore, the use of a compression coil spring can be considered advantageous. Without friction, the torque profiles would lie roughly in the middle between the two curves for extension and flexion. In the future, the replacement of the slide bearings of the revolute joint and the pulley with ball bearings can further reduce the hysteresis of the system.

The tests also show that the movement velocity has only a minor influence on the torque, as there is only dry friction and almost no damping in the system. Slightly increased/reduced torques during extension/flexion at five times the speed are probably due to inertia effects, for example, of the spring. However, they are negligibly small, so the averages of the 10°/s measurement provide a benchmark for all plausibly occurring velocities. It can also be observed that the support torque profiles at low SLs reach zero at a certain angle (>110°). At higher flexion angles, the torque would even become negative (not shown in Figure 5), but this effect is not relevant in practice as it will not affect the user negatively in everyday work. When the system is deactivated or at low SLs, it is not necessary to work with high flexion angles of over 100°. It would be possible to further reduce the support torque at SL 0% by making small design adjustments. However, this would also shift the angle of the zero crossing unfavourably to lower flexion angles. As the current 0% SL maximum torque is already low, counteracting only about 20% of the arm weight (assuming a 20 cm lever arm and a 5 kg arm), and is subjectively barely noticeable when worn, we assume that this is not necessary. With larger SLs, the theoretical zero crossing occurs only at angles above the maximum flexion angle (~ 155°). At this point at the latest, the support drops steeply to zero due to contact with a mechanical end stop. At the highest SL investigated, this drop already occurred at a lower angle. The reason for this was how the system was assembled, meaning that the cable was not fully tensioned at the maximum flexion angle and 100% SL. However, a different assembly procedure could easily rectify this in the future. At low angles, we observed another unintended effect. The support torque profiles deviate slightly from their monotonic decrease due to interference between the Bowden tubes and the arm lever. A greater torque must be applied in order to bend the Bowden tubes during extension movements below 30° (see *Figure 5*). Conversely, in the direction of flexion, the return forces of the Bowden tubes reduce the required torque. However, this effect only slightly influences the overall mechanical behavior and could be mitigated in the future by altering the routing of the Bowden tubes.

The results of the investigations on the support torque profiles appear plausible, and the observed effects can be explained well. The experimental setup delivers reproducible results, as can be seen from the repetition accuracy of the individual repetitions. An advantage is that no gravitational effects influence the torque curve. The contact of the arm lever and cantilever without relevant constraining forces also has a positive influence on the accuracy of the data. Nevertheless, it should be noted that there was certainly a minimal axis offset between the axes of the external drive and the revolute joint, which can slightly distort the absolute torque value. However, as the maximum relative displacement from the arm lever to the cantilever was always less than 2 mm, the influence on the measured torque is negligible. Also, the load cell itself could have caused a small measurement error. However, this error should be less than 0.2 Nm for this load cell and is therefore similarly irrelevant. Overall, we conclude that we created a precise torque measurement setup for exoskeletons and assume that measurements of our exoskeleton’s torque profiles were accurate.

Overall, we observed that the system provides high maximum torques (12 Nm) that are comparable to the strongest passive shoulder support exoskeletons available on the market (13 Nm [Watterworth et al., 2023]). The novel design of the adjustment mechanism, along with the resulting capability to integrate additional components (permanent magnet brakes, reset springs) into a repositioned drivetrain, has facilitated the development of a lightweight system. Consequently, the torque-to-weight ratio (2.1 Nm/kg) surpasses that of most active systems (1.0–1.2 Nm/kg [Rinaldi et al., 2023; Rossini et al., 2025]) and the limited number of semi-active approaches (1.2–1.3 Nm/kg [Grazi et al., 2020; Sänger et al., 2022; Otten, 2023]) and is even greater than that of some passive systems (1.6 Nm/kg [Pacifico et al., 2020]). However, most passive exoskeletons still exhibit higher ratios, reaching up to 6.7 Nm/kg (Maurice et al., 2020; Watterworth et al., 2023). We identified one active system with a comparable ratio to our approach: Ding et al. proposed a lightweight active exoskeleton (4.5 kg) capable of providing up to 10 Nm of support torque (torque-to-weight ratio: 2.2 Nm/kg, [Ding et al., 2024]). In fully active systems, the position of the exoskeleton relative to the user must be actively controlled via motors (Singer et al., 2020). Therefore, the motors need to be sufficiently fast to follow the user’s motions; otherwise, motion restrictions may occur. However, since the device was tested at maximum velocities of only ~110°/s (Ding et al., 2024), it remains uncertain whether the system can effectively track the user during faster movements. For instance, despite the Stuttgart Exo-Jacket’s capability to deliver shoulder actuator speeds of 240°/s, unpleasant delays were still observed (Singer et al., 2020). In our semi-active system, the fundamental support principle is passive, eliminating the need for active control of the motors to accommodate the user’s movements. Furthermore, we have demonstrated that this supporting torque is nearly independent of the user’s motion velocity, allowing the system to function effectively in highly dynamic situations. Additionally, compared to fully active approaches, the system does not require sensors or complex active control mechanisms, which can be advantageous in terms of robustness and cost.

### 5.2. Adjustment times

Linear functions can predict adjustment times with a high degree of accuracy and are essentially dependent on the adjustment distance (defined by the start and stop SL). The smallest measured adjustment of one SL takes about 0.5 s, a complete activation or deactivation about 1 s. Delay times of the pushbutton, potentiometer, and electronics are comparatively much smaller and can, therefore, be neglected. Minor deviations between the measured (validation measurement) and modelled adjustment times (0.4%), together with a high coefficient of determination (>99.9%), emphasize the predictive capability of the derived model. Neither the direction of motor motion nor the flexion angle of the exoskeleton had a relevant influence on the adjustment time. This is plausible, as the drive was dimensioned sufficiently large. The external torque on the motor changes depending on the angle and direction of motor motion and will therefore influence the current, but does not measurably slow down the acceleration or speed behaviour of the motor.

No quantitative comparison of the adjustment times could be made with other semi-active systems, as no empirical results for those metrics have been presented in the literature (Grazi et al., 2020; Sänger et al., 2022; Otten, 2023). However, given that the adjustment times range between 0.5 and 1 second, there should not be any significant delay after the user signals his intention (currently turning a switch). Therefore, we consider the exoskeleton to be fast for a semi-active exoskeleton. Higher motor accelerations, which would be easily adjustable, could further reduce these times without any hardware changes. However, such fast adjustments are probably not necessary for most activities. Therefore, slower and less powerful drives with the same rated torque could be used in the future to make the system lighter. It is also conceivable to increase the maximum support torque with a similar system weight if this is required for a specific activity. The current system may already be so fast that it can be used as a quasi-active system that adapts support on a second-by-second basis.

### 5.3. Energy consumption

Charge quantities were measured with a high repeatability. With an average standard deviation of less than 1 mAs, the deviation was always far less than 1% of the respective reference measurement. The exoskeleton requires only small amounts of charge to adjust the system (0.1–1.9 As). On this basis, long operating times can be expected even with the smallest batteries. However, this is reduced by the comparatively high continuous current of the electronic components, which is currently around 0.28 A. The charge required for adjustment strongly depends on the working angle and the start and stop SL. Dependencies on the adjustment distance seem intuitive, while the sometimes strong dependencies on the angle of the exoskeleton require further explanation. As previously mentioned, the drive unit of the exoskeleton has reset springs that are intended to reduce load peaks on the motor. They do this by providing additional resistance during the voluntary activation movements and supporting the return movement during power-intensive deactivation movements. This effect can also be seen in the amount of charge consumed. Charge quantities for activations can be predicted quite well with a linear relationship between start and stop SL and angle. The coefficient of the angle is an order of magnitude smaller than the other two (*Table 2*). It can, therefore, be concluded that the angle appears to have a smaller influence during activation. This can also be seen in Figure 6(b): At low angles, almost no change in charge consumption is recognizable. Here, only very small external forces act on the motor, so the charge consumption is primarily caused by the compression of the reset springs. The activation charge decreases slightly at higher angles. Here, the external force (from the spring mechanism in the spring colums) acts together with the motor against the reset springs, which is why less energy is required. The opposite is true for deactivation movements. At low angles, the external forces on the motor are low, so the reset springs are able to reset the motor on their own. Only a small amount of charge is required to supply the brake and control the position of the motor. At higher angles, the restoring force of the springs is no longer sufficient. The external forces increase, and the motor itself has to do more and more work. To summarize, the current required during activation is primarily influenced by the reset springs, whereas during deactivation, the external force and associated friction effects exert a greater influence. For deactivations, the relationship is strongly non-linear, which is why a linear approximation does not work so precisely here. The equations of the simple linear regression models also make it clear that activations are far less angle-dependent (smaller coefficient) than deactivations. These models are probably sufficient for a rough estimate of charge consumption and to illustrate effects. If a more precise estimate is required, the two detailed regression models should be used. We consider average deviations between model values and validation measurements of 6% and 17% (*R* ^2^ < 99%) to be sufficiently accurate to allow predictions of the charge consumed. Therefore, the models can be used to estimate the operating times of batteries. In order to enable a more accurate prediction model in the future, the individual influences of friction, damping and inertia in the system should be better understood, and a theoretical model should be set up instead of deriving a statistical model through empirical investigations, as is the case here.

However, the models should be accurate enough for a rough estimate of battery runtimes in simplified fictitious use cases. Although it is debatable whether the assumed cases would ever occur this way, they are suitable for quantifying the order of magnitude of operating times at low, high, and very high adjustment frequencies. In the case of low intensity (construction site), it can be assumed that a small 2 Ah power tool battery will last almost the whole day. For the dynamic use on an assembly line with overhead work, such a small battery should be changed in the middle of the working day. With an operating time of 2.2 hrs, the system can even be used in a quasi-active state, but a 4 Ah battery is recommended here to double the operating time so the battery can be changed midshift. The experiment shows the possible range of applications for the system. It can be used for less dynamic, semi-actively supported overhead activities but also as a quasi-active system in highly dynamic applications. Future investigations will show whether the latter offers benefits in practice. For most cases, we assume that a lower frequency of adjustments is sufficient for comfortable use. It should be noted that the basic energy consumption of the exoskeleton electronics is still quite high (~0.28 Ah per hour). In the less dynamic case 1, for example, the consumption of electronics accounts for a large proportion of the total energy consumed. If it were possible to halve the basic consumption of the electronics (e.g. by putting microcontrollers to sleep mode when not needed), the operating time in case 1 would increase from just under 7 hr to 13 hr. In the already highly dynamic case 2, a running time of almost 6 hr would be conceivable. This, in turn, opens up the possibility of using even smaller batteries, thereby reducing weight further. A runtime comparison with other active or semi-active systems could not be conducted, as the literature provides no or only rough estimates, without presenting measurement methods or empirical results for those metrics (Grazi et al., 2020; Sänger et al., 2022; Otten, 2023; Rinaldi et al., 2023; Ding et al., 2024; Rossini et al., 2025). However, we have demonstrated that the system exhibits overall low energy consumption, potentially allowing for long working times with only small and lightweight batteries (an entire day with just a 2 Ah battery), which should suffice for practical implementation.

## 6. Conclusion and outlook

In this work, a new semi-active exoskeleton for shoulder support was presented in detail and technical performance parameters were determined experimentally. The exoskeleton features a novel active support torque adjustment mechanism, enabling the design of a lightweight (5.8 kg, including the battery) and powerful exoskeleton, with most of its weight positioned on the user’s back and a slim overall design. Furthermore, the system exhibits a high maximum support torque (12 Nm), comparable to the strongest passive systems available on the market (Watterworth et al., 2023) and surpasses other active and semi-active systems designed for occupational shoulder support (Grazi et al., 2020; Sänger et al., 2022; Rinaldi et al., 2023; Ding et al., 2024; Rossini et al., 2025). Its overall torque-to-weight ratio is more favorable than that of most active and semi-active exoskeletons, and even some passive systems (e.g., Grazi et al., 2020; Pacifico et al., 2020; Sänger et al., 2022; Rinaldi et al., 2023; Rossini et al., 2025). The fundamentally passive support principle provides good mechanical transparency, as motion is always possible against a well-defined supporting spring resistance of the current SL, even in working situations involving rapid movements. Moreover, the support of the system can be continuously adjusted from maximum to (almost) no support, which enhances its versatility (e.g., for using tools of varying weights or working at different heights). The high adjustment speeds (~1 s for full activation/deactivation) also allow for dynamic adaptation of the provided support to the user’s needs, like fully active exoskeletons. Simultaneously, the exoskeleton’s low energy consumption enables continuous use of the system for several hours, potentially lasting an entire working day, with only a small battery.

In the future, several aspects of the current exoskeleton implementation could be improved to enhance its performance relative to its weight. This includes reducing friction in the system to minimize support torque hysteresis, changing the routing of Bowden tubes to eliminate resistance at low shoulder angles, optimizing electronics and control to reduce standby power consumption, and implementing smaller motors for less dynamic applications. Finally, we will conduct subject studies with the proposed exoskeleton to quantify the provided biomechanical relief and to evaluate the system’s robustness, usability, and comfort across various applications.

In addition to technical exoskeleton implementation, the test methodology proposed in this paper has proven capable of recording reproducible results on support torque profiles, adjustment times, and power consumption with a high degree of precision and repeatability. Hence, our methodology could be applied to investigate support torque profiles of different commercially available passive and active exoskeletons. For (semi-)active systems, metrics related to adjustment times and energy consumption could be examined to improve comparability among different devices.

## Data Availability

The data that support the findings of this study are available from the corresponding author upon reasonable request.
